# Enhanced UV Resistance and Improved Killing of Malaria Mosquitoes by Photolyase Transgenic Entomopathogenic Fungi

**DOI:** 10.1371/journal.pone.0043069

**Published:** 2012-08-17

**Authors:** Weiguo Fang, Raymond J. St. Leger

**Affiliations:** 1 Institute of Microbiology, College of Life Sciences, Zhejiang University, Hangzhou, Zhejiang, China; 2 Department of Entomology, University of Maryland, College Park, Maryland, United States of America; New Mexico State University, United States of America

## Abstract

The low survival of microbial pest control agents exposed to UV is the major environmental factor limiting their effectiveness. Using gene disruption we demonstrated that the insect pathogenic fungus *Metarhizium robertsii* uses photolyases to remove UV-induced cyclobutane pyrimidine dimers (CPD) and pyrimidine (6-4) photoproducts [(6-4)PPs] from its DNA. However, this photorepair is insufficient to fix CPD lesions and prevent the loss of viability caused by seven hours of solar radiation. Expression of a highly efficient archaeal (*Halobacterium salinarum*) CPD photolyase increased photorepair >30-fold in both *M. robertsii* and *Beauveria bassiana*. Consequently, transgenic strains were much more resistant to sunlight and retained virulence against the malaria vector *Anopheles gambiae*. In the field this will translate into much more efficient pest control over a longer time period. Conversely, our data shows that deleting native photolyase genes will strictly contain *M. robertsii* to areas protected from sunlight, alleviating safety concerns that transgenic hypervirulent *Metarhizium* spp will spread from mosquito traps or houses. The precision and malleability of the native and transgenic photolyases allows design of multiple pathogens with different strategies based on the environments in which they will be used.

## Introduction

The insect pathogenic fungi *Metarhizium robertsii* (formerly called *Metarhizium anisopliae*) [1] and *Beauveria bassiana* are being developed as biological control agents against agricultural pests [2], [3], and vectors of human diseases [4], [5], [6], [7], [8]. However, in spite of the environmental benefits of biological pesticides, few have been successfully commercialized due to inconsistent results compared with chemical insecticides. This is because of low virulence and short persistence outside the host, which is largely attributable to solar-UV radiation [9], [10], [11], [12], [13]. Efforts have been made to increase persistence by using UV-B protectants [12], or by spraying in the evenings [14]. In addition, strains have been engineered to express pigments which act as a sunscreen and increase UV-resistance 1.6-fold [15].

DNA is one of the major targets of solar ultraviolet (UV) radiation, and the resulting mutagenesis and cell death primarily involve the induction of chemical base modifications cyclobutane pyrimidine dimers (CPDs) and pyrimidine (6-4) photoproducts [(6-4)PPs] [16], [17]. Removal of these photolesions from the DNA is performed by the nucleotide excision repair (NER) pathway and by photoreactivation. NER is a complex multi-step process, involving the concerted action of ∼30 proteins that replace dimers by *de novo* synthesis. In contrast, photoreactivation is performed by one-enzyme photolyase pathways that monomerize dimers by using visible light as an energy source [18]. Notably, photolyases show substrate specificity for either CPDs (CPD photolyase) or 6–4PPs (6–4PP photolyase) [19].

Photoreactivation is the major mechanism for repairing UV-induced DNA damage in *Metarhizium* [20]. In this study, we functionally characterized *M. robertsii* CPD and (6–4)PP photolyases and found that the (6–4)PP photolyase effectively removed the (6-4)PPs lesions introduced by sunlight, whereas CPDs accumulated causing cytotoxic effects and loss of virulence. As expressing a plant photolyase reduced mutations in the UV-exposed skin of mice [18], and overexpressing a native photolyase moderately increased growth of UV-treated *Arabidopsis thaliana* [21], we decided to investigate the extent to which photolyases could be used to engineer UV-tolerance in entomopathogens. We found that overexpressing the native *M. robertsii* photolyase or expressing the photolyase of a highly UV tolerant *Halobacterium* [22], both imparted increased UV tolerance, but the *Halobacterium* enzyme was much more effective and achieved a 32-fold improvement in survivability to sunlight. Unlike wild type strains, *M. robertsii* or *B. bassiana* expressing *Halobacterium* photolyase retained virulence against the malaria vector *Anopheles gambiae* even after several hours exposure to high UV sunlight. In the field, this improved persistence should translate into much more effective pest control over a longer time frame.

We recently developed hypervirulent transgenic strains that applied in houses or mosquito traps have great potential for controlling mosquitoes and malaria parasites [4], [7], [8], [23]. The corollary of seeking to increase persistence of strains with wild type levels of virulence is the perceived regulatory or environmental need to contain these first generation transgenic products. UV-hypersensitive mutants could be immediately useful for hypervirulent transgenic strains which are not designed to recycle and/or are to be applied in enclosures. Most current anti-malarial programs, including those involving *Metarhizium*, target mosquitoes in houses where the pathogen would be protected against direct sunlight [6], [24].

## Results and Discussion

### Characterization of the CPD and (6-4)PP photolyases in *Metarhizium robertsii*

We identified two putative photolyases (Maa05216 and Maa05870) in the *Metarhizium* genome [25]. Maa05216 encodes a 587-aa protein, designated as MrPHR1 (Genbank accession number: JN694761), which is highly similar to functionally characterized CPD photolyases of *Trichoderma atroviride* (84%) [26], *Neurospora crassa* (78%) [27] and *Saccharomyces cereviase* (55%) [28]. To use sunlight for DNA repair, photolyases need a catalytic reduced flavin-adenine dinucleotide (FAD) chromophore and a light harvesting chromophore which is either methyl tetrahydrofolate or another flavin [26]. The light harvesting and catalytic domains of MrPHR1 are similar to those of bacteria (>45%), and not to sequences in *Drosophila* and *Arabidopsis* (Fig. S1). Maa05870 encodes a 526-aa protein designated MrPHR2 (Genbank accession number: JN694762). MrPHR2 showed significant similarity to (6-4)PP photolyases of *Arabidopsis* (51%) [29], and a putative (6-4)PP photolyase from the fungus *Cercospora zeae-maydis* (71%) [30].

To investigate the biological functions of the putative *Metarhizium* CPD (MrPHR1) and (6–4)PP (MrPHR2) photolyases, we constructed three mutants: (i) *Mrphr1*-disruption mutant *ΔMrphr1*, (ii) *Mrphr2*-disruption mutant *ΔMrphr2,* and (iii) *Mrphr1* and *Mrphr2*-double disruption mutant *ΔMrphr1ΔMrphr2*. *ΔMrphr1*and *ΔMrphr2* were complemented by transformation with *Mrphr1* and *Mrphr2,* respectively, as described in Materials and Methods. The complemented strains did not differ from their parent wild type in any of our experiments. Because of insufficient selection markers for the complementation of the double mutant *ΔMrphr1ΔMrphr2*, we analyzed five randomly selected *ΔMrphr1ΔMrphr2* mutants to confirm that changes results from the simultaneous disruption of *Mrphr1* and *Mrphr2*.

Photolyases are expected to allow lesion-specific repair of CPDs or (6-4)PPs upon exposure to photoreactivating light. We investigated light-dependent removal of photolesions with an ELISA assay using antibodies specific for either CPDs or (6-4)PPs (Cosmo Bio Co. LTD. Japan). The parental wild type strain and the three mutants were irradiated with a modest dose of UV radiation (8 mW/cm^2^) and, as expected, a strong ELISA signal was detected with both antibodies, while non-irradiated cells did not contain CPDs and (6-4)PPs. Thus, photolesions are immediately induced by UV exposure. Photoreactivation (4 h) of the UV-treated wild type strain reduced CPDs by 60% and (6-4)PPs by 37%. Photoreactivation did not remove CPDs in *ΔMrphr1* and (6-4)PPs in *ΔMrphr2,* confirming that MrPHR1is a CPD photolyase and MrPHR2 is a (6-4)PP photolyase (Table S1).

We then checked germination rates following exposure to a range of UV doses and photoreactivating light. At low doses (6 mW/cm^2^), UV significantly delayed the germination of *ΔMrphr1ΔMrphr2,* and to a lesser extent *ΔMrphr1* (P<0.01), but had no impact on *ΔMrphr2*. A UV dose (10 mW/cm^2^) sufficient to slow germination of *ΔMrphr2* still impacted *ΔMrphr1ΔMrphr2* to a significantly (P<0.01) greater extent than either *ΔMrphr1* or *ΔMrphr2*. A high UV dose (30 mW/cm^2^) completely inactivated *ΔMrphr1ΔMrphr2* and *ΔMrphr1* spores, whereas 23% of *ΔMrphr2* spores and 50% of wild type spores remained viable. MrPHR1 and MrPHR2 therefore work synergistically during photoreactivation to repair UV-induced damage to DNA (Table 1).

**Table 1:** Germination rates of spores of *ΔMrphr1, ΔMrphr2, ΔMrphr1ΔMrphr2* and the wild type irradiated by UV followed by either photoreactivation or NER.

UV dose (mW/cm^2^)	Germination Rate % following treatment and 24 or 48 h incubation at 27°C							
	24 h incubation				48 h incubation			
	WT	*ΔMrphr1*	*ΔMrphr2*	*ΔMrphr1ΔMrphr2*	WT	*ΔMrphr1*	*ΔMrphr2*	*ΔMrphr1ΔMrphr2*
Treatment 1: 4 h Photoreactivation and 24 or 48 h incubation at 27°C								
0	100	100	100	100	100	100	100	100
6	89.6±2.1	25.4±2.2	88.4±1.2	10.5±1.1	100	78.7±2.8	99.2±0.5	55.1±2.2
10	83.2±2.3	6.4±0.6	69.8±2.1	4.4±0.5	100	71±2.5	97.2±1.2	35.3±2.2
20	29.2±1.9	0.1±0.02	2.6±0.09	0	58.6±2.7	11.2±1.5	40.9±1.7	5.4±0.8
30	9.6±0.7	0	0	0	49.6±2.5	0	23.3±1.1	0
Treatment 2: 4 h incubation in the dark (NER)								
0	100	100	100	100	100	100	100	100
6	38.8±2.4	25.1±2.1	23.5±2.1	11.1±0.5	69.8±1.7	65.1±1.3	66.0±1.6	59.5±2.5
10	27.4±1.1	5.2±0.9	11.9±1	2.7±0.07	71±2.3	51±2.5	53.1±2.8	38.9±3.2
20	3.3±0.06	0	0	0	0	0	0	0
30	0	0	0	0	0	0	0	0

Cells kept in the dark would lack the energy source required for photolyase activity, and thus removal of photolesions would presumably be due to NER. When treated with UV (10 mW/cm^2^) and placed immediately in the dark (48 hours), about 52% of *ΔMrphr1* or *ΔMrphr2* spores germinated, which was significantly (P<0.01) less than the wild type (71%), and significantly (P<0.01) more than *ΔMrphr1ΔMrphr2* (39%) (Table 1). This light-independent removal of photolesions demonstrates that photolyases enhance NER. Photolyases also facilitate NER in plants and yeast by an incompletely understood mechanism [31]. However, as expected, photoreactivation significantly (P<0.05) increased the germination rates of the wild type strain and single disruption mutants (*ΔMrphr1* and *ΔMrphr2*), whereas the impact of photoreactivation on *ΔMrphr1ΔMrphr2* did not reach statistical significance (P = 0.09) (Table 1).

The virulence of *ΔMrphr1*, *ΔMrphr2* and *ΔMrphr1ΔMrphr2* against adult mosquitoes (*Anopheles gambiae*) was the same as that of the wild type, with LT_50_ values (time taken to kill 50% of the insects) of approximately 5.5 days.

### CPD production resulting from solar-radiation outstripped the CPD repair capacity of wild type *M. robertsii*

**Figure 1:**
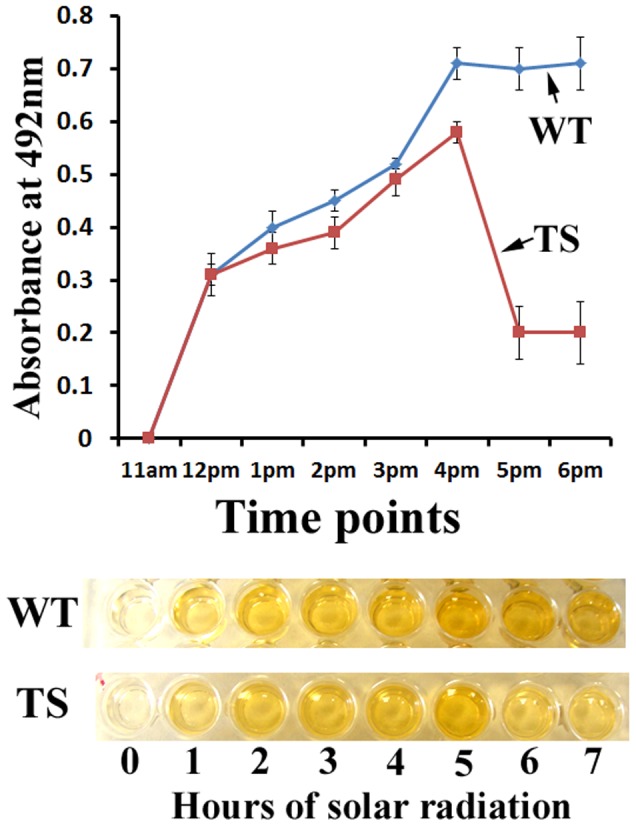
Quantification of CPDs in solar-irradiated mycelia with ELISA. Upper panel: the amount of CPDs in wild type and transgenic *M. robertsii* expressing a CPD photolyase (HsPHR2). Down panel: a representative ELISA assay plate using CPD monoclonal antibody. WT: wild type; TS: a transgenic strain expressing HsPHR2.

Next, we studied whether MrPHR1 and MrPHR2 provided protection against natural sunlight. Seven hours-exposure (from 11am to 6pm) killed ∼60% of *ΔMrphr1* and *ΔMrphr1ΔMrphr2* spores, and slowed germination of *ΔMrphr2* and the wild type ∼3-fold. This is consistent with CPDs having greater cytotoxic effects on *M. robertsii* than (6-4)PPs. A time course study showed that CPDs increased continually in wild type *M. robertsii* from 11am to 4pm and then plateaued (Fig. 1). (6-4)PPs were undetectable at any time point suggesting that the 6–4PP photolyase activity of MrPHR2 kept pace with the formation of (6-4)PPs. We concluded that CPDs are the major UV-induced photoproduct in *M. robertsii,* and the activity of MrPHR1 is insufficient to repair this damage.

### Overexpressing CPD photolyases increased the tolerance of *M. robertsii* to UV radiation

**Figure 2:**
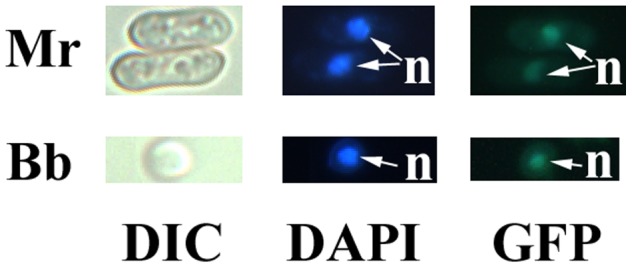
The effectiveness of the *Metarhizium* transcription factor (maa08770) nuclear localization signal peptide (NLS) at directing green fluorescent protein (GFP) into fungal nuclei. Upper row: *M. robertsii*. Lower row: *B. bassiana*. DIC: Differential interference contrast; DAPI: nuclear DNA was stained with DAPI (blue, 4′,6′-diamidino-2-phenylindole); GFP: green fluorescent signal in nuclei; n: nucleus.

We generated *M. robertsii* strains overexpressing its native CPD photolyase MrPHR1 or the CPD photolyase HsPHR2 from the extremophile archaea *Halobacterium salinarum* [22]. HsPHR2's extremely efficient photoreactivation activity allows *H. salinarum* to tolerate very high levels of solar-radiation [22]. To direct the archaeal photolyase to the nuclear DNA, a nuclear localization signal peptide (NLS) from *Metarhizium* was fused to HsPHR2. The putative NLS of transcription factor maa08770 was identified using WoLF PSoRT (http://wolfpsort.org). To test the effectiveness of the maa08770 NLS, we constructed a transgenic *M. robertsii* strain that constitutively expressed the fusion protein NLS:GFP (enhanced green fluorescence protein fused to the C-terminus of the NLS). Green fluorescence was visible in the nuclei only (Fig. 2).

HsPHR2 was then fused to the C-terminus of the NLS, generating NLS:HsPHR2. The coding sequence of NLS:HsPHR2 terminated with a hexa histidine-tag and preceded by a start codon (ATG) was synthesized commercially by Genscript (NJ. USA) using the preferred codon usage of *M. robertsii,* and cloned downstream of the constitutive glyceraldehyde-3-phosphate dehydrogenase promoter (*Pgpd*) of *Aspergillus nidulans* [32]. The *M. robertsii* CPD photolyase gene *Mrphr1* was also hooked up with a hexa histidine-tag and *Pgpd*. Insertion of *gpd:NLS:HsPhr2* or *gpd:Mrphr1* into the *M. robertsii* genome was confirmed with Southern blots (single-copy transformants were selected for subsequent analyses), and immunoblot analysis of protein extracts using rabbit anti-His tag polyclonal antibody (Anaspec, CA USA) showed bands of the expected sizes for NLS:HsPHR2 and MrPHR1 (Anaspec, CA USA) (Fig. S2).

We tested the effects of UV on germination of the wild type strain and the transformants Mr-HsPHR2 (expressing HsPHR2) and Mr-OMrPHR1 (overexpressing MrPHR1). In the absence of UV, Mr-HsPHR2 and Mr-OMrPHR1 had wild type germination rates. After a dose of UV (20 mW/cm^2^) and 4 h photoreactivation, 59% of wild type spores were viable, which was significantly less than Mr-OMrPHR1 (83%)(*P*<0.05) which in turn was significantly (*P*<0.05) less than Mr-HsPHR2 (100%)(Table 2). However, without photoreactivation, UV-radiated spores of Mr-HsPHR2 and Mr-OMrPHR1 had wild type germination rates (Table 2), suggesting that the additional CPD photolyase activity did not enhance *M. robertsii's* NER.

**Table 2:** Germination rates of spores of wild type and transgenic *M. robertsii* radiated by UV (followed by either photoreactivation or NER) and by sunlight.

	Germination Rate % following treatment and 24 or 48 h incubation at 27°C					
	24 h incubation			48 h incubation		
	Mr-WT	Mr-OMrPHR1	Mr-HsPHR2	Mr-WT	Mr-OMrPHR1	Mr-HsPHR2
Treatment 1: UV radiation (mW/cm^2^) followed by 4 h photoreactivation						
0	100	100	100	100	100	100
10	83.2±2.3	98.3±1.1	99.2±0.5	100	100	100
20	29.2±1.9	76.2±2.9	88.5±1.3	58.6±2.7	82.5±2.1	100
30	9.6±0.7	15.6±1.1	25.4±2.1	49.6±2.5	56.5±1.3	68.2±2.7
Treatment 2: UV radiation (mW/cm^2^) followed by 4 h incubation in the dark (NER)						
0	100	100	100	100	100	100
10	27.4±1.1	26.9±1.3	27.2±2.1	71±2.3	68±2.5	71±2.9
20	3.3±1.1	3.2±0.9	3.9±0.8	0	0	0
30	0	0	0	0	0	0
Treatment 3: solar radiation (h)						
2	100	ND	100	100	ND	100
4	48.8±1.3	ND	56±2.1	100	ND	100
6	4.6±0.8	ND	45.2±1.3	100	ND	100
7	1.1±0.2	ND	35.4±1.5	100	ND	100

CPDs were quantified following treatment with UV (30 mW/cm^2^) and photoreactivation. Both transgenic strains were significantly more efficient than the wild type at eliminating CPDs between 1 h to 4 h photoreactivation. At 4 h, the wild type and Ma-OMrPHR1 had 6.9-fold and 2.4 -fold more CPDs, respectively than Mr-HsPHR2 (Table S2). Collectively, this data shows that the increased tolerance of Mr-HsPHR2 and Ma-OMrPHR1 to UV radiation is due to improved CPD-repair ability, with HsPHR2 being more effective than MrPHR1.

### Expressing HsPHR2 increased the tolerance of *M. robertsii* to sunlight

We next confirmed that HsPHR2 protects *Metarhizium* from solar radiation. The amount of CPDs in WT mycelium exposed to sunlight increased continually between 11am and 4 pm, and then plateaued. However, CPD levels in Mr-HsPHR2 decreased sharply from 4pm and by 5pm, the wild type had 3.5-fold more CPDs than Mr-HsPHR2 (Fig. 1). After 7 h solar-radiation, and 24 h in the dark, the germination rate of Mr-HsPHR2 spores (35.4%) was 32-fold higher than that of the wild type strain (1.1%).

**Figure 3:**
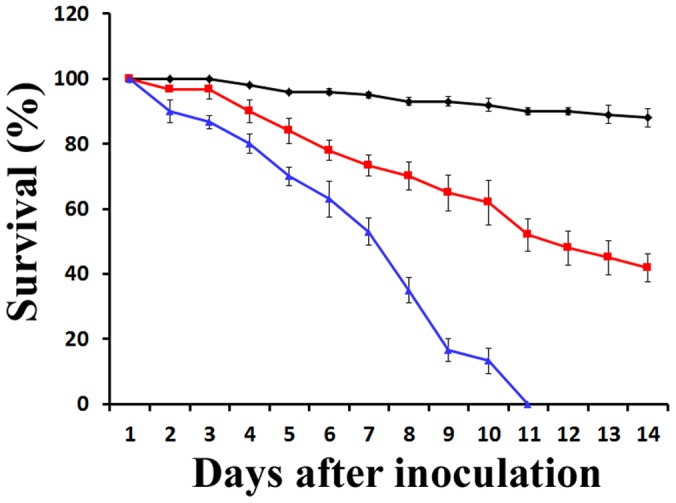
Kinetics of mosquito survivorship in bioassays. Adult female mosquitoes (*A. gambiae*) were sprayed with *M. robertsii* spore suspensions (1×10^7^ spores/mL) that had been irradiated by sunlight for 7 h. Red = wild type *M. robertsii*; Blue = *M. robertsii* expressing a CPD photolyase (HsPHR2) from *H. salinarum*; black = control insects that were treated with 0.05% Tween-80.

The virulence of solar-radiated spores was tested against adult *Anopheles gambiae*. Solar-radiated (7 hours) wild type spores took 14 days to kill 58% of mosquitoes, whereas solar-radiated Mr-HsPHR2 spores killed 100% of mosquitoes within 11 days (*P*<0.05). Without solar radiation, Mr-HsPHR2 and the wild type killed 100% of mosquitoes within 9 days. Nine days is significantly faster than 11 days (P<0.05) (Fig. 3), but the data shows that expression of HsPHR2 reverses much of the impact of solar radiation on virulence.

### Expressing HsPHR2 also increased UV-tolerance of *B. bassiana*

Solar radiation is a major barrier to successful exploitation of *B. bassiana* (34 Fernandes), so we transformed *B. bassiana* with HsPHR2 to determine if this is a generally applicable method for enhancing UV tolerance. Following confirmation that the *M. robertsii* nuclear localization signal directed GFP into *B. bassiana* nuclei (Fig. 2), we then transformed *gpd:nls: Hsphr2* into *B. bassiana* to produce Bb-HsPHR2. Eighty one % of Bb-HsPHR2 spores remained viable following UV-radiation (10 mW/cm^2^) and photoreactivation (4 h), compared to only 43% of wild type spores (p<0.01). In contrast to *M. robertsii,* the viability of Bb-HsPHR2 spores (51.5%) was significantly higher than wild type (20.4%) (*P*<0.01) even without photoreactivation, showing that HsPHR2 enhances photolesion repair by NER in *B. bassiana* (Table 3).

**Table 3:** Germination rates of spores of wild type *B. bassisana* and the transgenic strain Bb-HsPHR2 radiated by UV (followed by either photoreactivation or NER) and by sunlight.

	Germination Rate % following treatment and 24 or 48 h incubation at 27°C			
	24 h incubation		48 h incubation	
	Bb-WT	Bb-HsPHR2	Bb-WT	Bb-HsPHR2
Treatment 1: UV radiation (mW/cm^2^) followed by 4 h photoreactivation				
0	100	100	100	100
10	30.8±1.3	51±2.1	43±1.6	81±2.6
20	15.2±1.2	34±2.3	25±2.1	50.4±2.9
30	4.4±0.8	15.0±1.1	8.3±0.7	21.2±1.3
Treatment 2: UV radiation (mW/cm^2^) followed by 4 h incubation in the dark (NER)				
0	100	100	100	100
10	12.5±0.9	39.8±2.1	20.4±2.7	51.5±2.5
20	0	0	0	12.7±0.4
30	0	0	0	0
Treatment 3: solar radiation (h)				
2	81.1±1.5	94.1±1.1	92.5±2.6	100
4	8.5±0.8	86.5±5.2	10±0.9	100
6	1.2±0.3	19.9±1.9	5.9±1.2	38±3.1
7	0.3±0.02	18.7±1.9	0.7±0.03	31±3.5

*B. bassiana* spores expressing HsPHR2 were also much more tolerant to solar radiation. One hundred percent of Bb-HsPHR2 spores survived 4 hours of sunlight, as compared to 10% of the wild type, and 31% survived 7 hours exposure, a 44-fold increase compared to the wild type (0.7%). Non-solar-radiated Bb-HsPHR2 and wild type spores were equally viable (P = 0.31). Similar to *M. robertsii*, quantities of CPDs in the mycelium of wild type *B. bassiana* increased from 11 am until 6pm, while in Bb-HsPHR2, CPD levels declined sharply after 4pm. By 6pm, CPD levels were 4.6-fold higher in the wild type than in Bb-HsPHR2 (Fig. S3).

Expression of PHR2 also alleviated the attenuation of *B. bassiana*'s virulence by solar-radiation. *B. bassiana* spores exposed to 4 h sunlight did not kill *A. gambiae*, while similarly treated Bb-HsPHR2 spores caused 42% mortality in two weeks. Bb-HsPHR2 exposed to two hours of sunlight retained wild type levels of virulence (P = 0.55), and killed 100% of mosquitoes in 15 days, whereas similarly treated wild type spores caused only 60% mortality (Fig. S4).

## Conclusion

The precision and malleability of the photolyases allows design of multiple pathogens with different strategies. To date, it has been axiomatic that a recombinant pathogen should not persist and reproduce in the field. All microbial agents are inactivated by exposure to sunlight, and photoinactivation is the major environmental factor limiting their effectiveness in field conditions. UV-hypersensitive strains with deleted photolyases will provide additional containment and could be used to prevent a pathogen surviving outside an enclosure or the host. Many insect pathogens already show a high degree of specificity, and signal transduction pathways have been identified that could be used to develop recombinant microbes that show very narrow specificity for target pests e.g., anophelenes or culicines or ticks [25]. A specific fungus that can utilize a transgenic photolyase to persist in the environment could provide sustainable cheap control for much longer periods than existing chemicals. Producing such an organism could not have been envisioned as recently as a decade ago. However, we now have the molecular biological knowledge and techniques that will make creation of these microbes highly feasible.

## Materials and Methods

### Fungal and bacterial strains

*Beauveria bassiana* strain Bb09 and *M. robertsii* ARSEF2575 were cultured as previously described [33]. *E. coli* DH5α was used for plasmid construction. *Agrobacterium tumefaciens* AGL1 was used for fungal transformation.

### Gene Cloning and Disruption

To construct the *Mrphr1* disruption plasmid pDMrphr1, the 5′-end and 3′-end of *Mrphr1's* ORF were cloned by PCR and inserted into the Xba I and Spe I sites, respectively, of the plasmid pFBARGFP [34]. Similarly, the 5′-end and 3′-end of *Mrphr2* ORF were inserted into the EcoRV and XbaI sites, respectively, of the plasmid pPK2SurGFPD [35], producing the *MrPhr2* disruption plasmid pDMrphr2. The disruption mutants (*ΔMrphr1* and *ΔMrphr2*) were obtained utilizing *A. tumefaciens*. To obtain a mutant disrupted in both *Mrphr1* and *Mrphr2*, *Mrphr2* was disrupted in *ΔMrphr1* using the plasmid pDMrphr2. The plasmids pFBARGFP with genomic DNA of Mrphr2 and pPK2SurGFPD containing genomic Mrphr1 were transformed into *ΔMrphr2* and *ΔMrphr1*, respectively, for complementation. The primers employed in this study and their usages are given in Table S3.

### Characterization of a nuclear localization sequence of *M. robertsii*

PSORT II [36] was used to identify nuclear localization sequences from 20 putative transcription factors of *M. robertsii* [25], and a peptide (DKKRKSWGQVLPEPKTNLPPRKRAKT), designated as NLS, from the transcription factor maa08770 was chosen for functional analysis. The fusion protein NLS:GFP was constructed to test the ability of NLS to direct a protein into nuclei of *M. robertsii* and *B. bassiana*. A DNA fragment containing the translation start site (ATG), the coding sequence of NLS and the coding sequence of the five N-terminal amino acids of GFP was synthesized and used as a forward primer to amplify the DNA fragment coding NLS:GFP with a reverse primer designed according to the C-terminus of GFP. The plasmid pFBARGFP was used as DNA template for the PCR reaction [34]. The PCR product was cloned into pGEM-T (Promega) and sequenced. The coding sequence of NLS:GFP was excised from pGEM-T and inserted downstream of the constitutive promoter of Pgpd in pBARGPE1 to produce Pgpd:NLS:GFP. The NLS:GFP expression cassette was then moved to pFBARGFP to replace the GFP cassette and form the expression plasmid pNLS:GFP. *A. tumefaciens* AGL-1 was used to mediate transformation of the expression plasmid into *M. robertsii* and *B. bassiana* as previously described [37].

### Expressing an archaeal CPD photolyase in *B. bassiana* and *M. robertsii*

The coding sequence of *H. salinarum* CPD photolyase HsPHR2 preceded by the coding sequence of NLS and terminated with a hexa histidine-tag was synthesized commercially (Genscript, NJ. USA), using the preferred codon usage of *M. robertsii* (http://www.kazusa.or.jp/codon/). A translation start site (ATG) was added immediately before the NLS coding sequence. The coding sequence of the fusion protein NLS:HsPHR2 was inserted into BamH I and EcoR V sites downstream of Pgpd in the plasmid pBARGPE1. The NLS:HsPHR2 expression cassette was then moved into pFBARGFP to form the expression plasmid pNLS:HsPHR2, which was transformed into fungi using *A. tumefaciens*.

The insertion of the NLS:HsPHR2 expression cassette into fungal genomes was confirmed by Southern blotting analysis. The Dig-labeled (Roche) *Pgpd* was used as probe. The expression of NLS:HsPHR2 was investigated by Western blot analysis using Rabbit anti-His tag polyclonal antibody (Anaspec, CA USA). Putative transformants were grown in SDB for 48 h, and the total protein from mycelium was extracted for Western blot analysis.

### Quantification of CPDs and (6-4)PPs in mycelium

Fungal genomic DNA was isolated with the DNeasy Plant Mini kit (Qiagen CA), and the concentration of DNA was quantified using a spectrometer (Biophotometer, Eppendorf, USA). DNA was denatured and applied into 96 well microliter plates (Polyvinylchloride flat-bottom, Thermo, Milford, MA). Either 10 ng (for CPD assay) or 400 ng of DNA [for (6-4)PP assay] in 50 µl of PBS was applied in each well and monoclonal antibodies against CPD and (6-4)PP were used for quantification by ELISA as described in manuals (Cosmo Bio Co., LTD., Japan). The absorbance at 492 nm of each well was determined by a SpectraMax Plus 384 Absorbance Microplate Reader (Molecular Device Inc. CA USA). Three replicates were used for each sample.

### Treatment of spores and mycelium with UV-radiation and solar-radiation

Unless mentioned otherwise, the germination behavior of UV-irradiated *B. bassiana* and *M. robertsii* spores (5×10^5^) was examined in 3 ml of 0.01% yeast extract per Petri dish (diameter = 3 cm).

Petri dishes were irradiated in a UV-B GS GENE Linker UV Chamber (Bio-Rad) and then either exposed to light energy (photoreactivation) by placing the dishes 60 cm from two fluorescent lights bulbs (15 W, Sylvania F15T8/CW/SS) or kept in the dark by covering immediately with aluminum foil. Spores that had not germinated after 48 h incubation are considered unviable as described [20].

To obtain solar-irradiated spores, 30 ml of a spore suspension (10^7^ spores/ml) in 0.01% Tween-80 plus 0.01% yeast extract was placed in a Petri dish (diameter = 90 mm) against a light-colored background and exposed to sunlight for up to 7 hours (11am to 6pm) with constant gentle shaking. The temperature ranged between 22 and 30°C. Fresh tween solution was added as necessary to compensate for loss by evaporation. The days chosen were cloudless with a midday UV index of 9 [incident power density of 0.6 mW/(nm m^2^) at 295 nm, 74 mW/(nm m^2^) at 305 nm and 478 mW/(nm m^2^) at 325 nm]. After treatment, the dishes were either kept in the dark for germination analysis, or spores were pelleted by centrifugation and suspended in 30 ml of the tween solution for bioassay as described below.

To quantify photoproducts in mycelium treated with UV or solar-radiation, one milliter of spore suspension (10^7^ spores per ml) was added to 25 ml of SDB in a Petri dish (diameter = 90 mm), and incubated at 27°C for 48 h. The SDB medium was then removed by pipetting and the mycelium was washed with sterile distilled water 3 times. The mycelium was then incubated in 25 ml of 0.01% yeast extract followed by UV-radiation, solar-radiation, photoreactivation or dark treatment as described for spores.

### Bioassay

Fungal virulence was assayed against adult mosquitoes (*A. gambiae*) as previously described [4]. The *SPSS* program was used to calculate the LT_50's_ (time taken to kill 50% of mosquitoes).

## Supporting Information

Figure S1 Phylogenetic analysis of photolyase domain (A) and FAD domain (B) of MrPHR1 and their homologs from bacterium, archae, insect and plant. MEGA5 software was used to carry out the analysis. Bootstrap values are adjacent to each internal node, representing the percentage of 1,000 bootstrap replicates.
(PDF)

Figure S2 Confirmation of the expression of *H. salinarum* photolyase HsPHR1 in *M. robertsii* and *B. bassiana*. (**A**) Southern blot analysis confirming insertion of *Pgpd:nls:HsPhr1* in the genomes of *Metarhizium* and *Beauveria*. Genomic DNA was digested with *EcoR* I and *Spe* I. The promoter *Pgpd* was used as a probe. 1: wild type *M. robertsii*; 2: a *Metarhizium* transformant expressing NLS:HsPHR1; 3: a *Beauveria* transformant expressing NLS:HsPHR1; 4: wild type *B. bassiana*. (**B**) Detection of *M. robertsii* or *B. bassiana* expressed NLS:HsPHR1 with Western blot analysis using rabbit anti-His-tag antibodies. Mycelium (0.1 g wet weight) grown in SDB culture was used for protein preparation, and 20 µg of protein was loaded in each lane. 1: a *Metarhizium* transformant expressing NLS:HsPHR1; 2: wild type *M. robertsii*; 3: a *Beauveria* transformant expressing NLS:HsPHR1; 4: wild type *B. bassiana*.
(PDF)

Figure S3 The amount of CPDs in the solar-irradiated mycelium of wild type and transgenic *B. bassiana* expressing a CPD photolyase (HsPHR2) from *H. salinarum*. The quantification of CPDs was performed by ELISA with CPD monoclonal antibody. Blue: wild type; Orange: a transgenic strain expressing HsPHR2.
(PDF)

Figure S4 Kinetics of mosquito survivorship in bioassays. Female adult mosquitoes (*A. gambiae*) were sprayed with *B. bassiana* spore suspensions (1×10^7^ spores/mL) that were irradiated by sunlight for 4 h. Red = the wild type strain; Blue = a transgenic strain expressing a CPD photolyase (HsPHR2) from *H. salinarum*; black = control insects that were treated with 0.05% Tween-80.
(PDF)

Table S1 The ability to repair CPDs and (6-4)PPs of *ΔMrphr1, ΔMrphr2, ΔMrphr1ΔMrphr2* and the wild type irradiated by UV followed by either photoreactivation or NER.
(PDF)

Table S2 The ability to repair CPDs of wild type and transgenic *M. robertsii* and *B. bassiana* radiated by UV followed by photoreactivation.
(PDF)

Table S3 The sequences of primers and synthesized genes used in the study.
(PDF)
