# Supplementary material for: Enhanced UV Resistance and Improved Killing of Malaria Mosquitoes by Photolyase Transgenic Entomopathogenic Fungi
Source: PLoS One. 2012 Aug 17;7(8):e43069. doi: 10.1371/journal.pone.0043069 (PMC3422317; doi:10.1371/journal.pone.0043069)
Supplement: Figure S1 — Phylogenetic analysis of photolyase domain (A) and FAD domain (B) of MrPHR1 and their homologs from bacterium, archae, insect and plant. MEGA5 software was used to carry out the analysis. Bootstrap values are adjacent to each internal node, representing the percentage of 1,000 bootstrap replicates. (PDF) [file pone.0043069.s001.pdf]

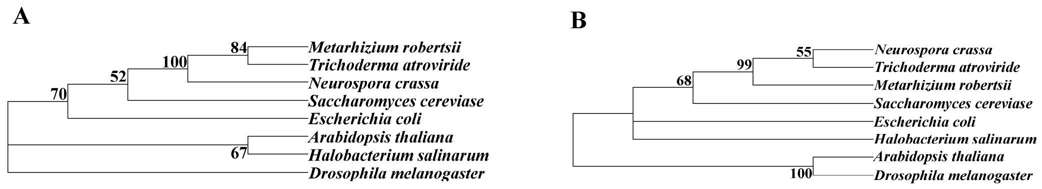

Fig. S1 Phylogenetic analysis of photolyase domain (A) and FAD domain (B) of MrPHR1 and their homologs from bacterium, archae, insect and plant. MEGA5 software was used to carry out the analysis. Bootstrap values are adjacent to each internal node, representing the percentage of 1,000 bootstrap replicates.
