# Supplementary material for: Enhanced UV Resistance and Improved Killing of Malaria Mosquitoes by Photolyase Transgenic Entomopathogenic Fungi
Source: PLoS One. 2012 Aug 17;7(8):e43069. doi: 10.1371/journal.pone.0043069 (PMC3422317; doi:10.1371/journal.pone.0043069)
Supplement: Figure S2 — Confirmation of the expression of H. salinarum photolyase HsPHR1 in M. robertsii and B. bassiana. (A) Southern blot analysis confirming insertion of Pgpd:nls:HsPhr1 in the genomes of Metarhizium and Beauveria. Genomic DNA was digested with EcoR I and Spe I. The promoter Pgpd was used as a probe. 1: wild type M. robertsii; 2: a Metarhizium transformant expressing NLS:HsPHR1; 3: a Beauveria transformant expressing NLS:HsPHR1; 4: wild type B. bassiana. (B) Detection of M. robertsii or B. bassiana expressed NLS:HsPHR1 with Western blot analysis using rabbit anti-His-tag antibodies. Mycelium (0.1 g wet weight) grown in SDB culture was used for protein preparation, and 20 µg of protein was loaded in each lane. 1: a Metarhizium transformant expressing NLS:HsPHR1; 2: wild type M. robertsii; 3: a Beauveria transformant expressing NLS:HsPHR1; 4: wild type B. bassiana. (PDF) [file pone.0043069.s002.pdf]

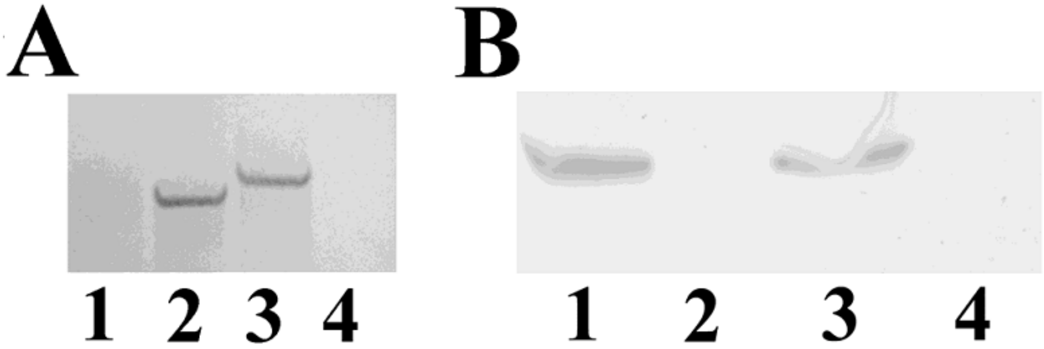

Fig. S2 Confirmation of the expression of *H. salinarum* photolyase HsPHR1 in *M. robertsii* and *B. bassiana*.(**A**) Southern blot analysis confirming insertion of *Pgpd:nls:HsPhr1* in the genomes of *Metarhizium* and *Beauveria*. Genomic DNA was digested with *EcoR* I and *Spe* I. The promoter *Pgpd* was used as a probe. 1: wild type *M. robertsii*; 2: a *Metarhizium* transformant expressing NLS:HsPHR1; 3: a *Beauveria* transformant expressing NLS:HsPHR1; 4: wild type *B. bassiana*. (**B)** Detection of *M. robertsii* or *B. bassiana* expressed NLS:HsPHR1 with Western blot analysis using rabbit anti-His-tag antibodies. Mycelium (0.1g wet weight) grown in SDB culture was used for protein preparation, and 20 μg of protein was loaded in each lane. 1: a *Metarhizium* transformant expressing NLS:HsPHR1; 2: wild type *M. robertsii*; 3: a *Beauveria* transformant expressing NLS:HsPHR1; 4: wild type *B. bassiana*.
