# Supplementary material for: Enhanced UV Resistance and Improved Killing of Malaria Mosquitoes by Photolyase Transgenic Entomopathogenic Fungi
Source: PLoS One. 2012 Aug 17;7(8):e43069. doi: 10.1371/journal.pone.0043069 (PMC3422317; doi:10.1371/journal.pone.0043069)
Supplement: Figure S3 — The amount of CPDs in the solar-irradiated mycelium of wild type and transgenic B. bassiana expressing a CPD photolyase (HsPHR2) from H. salinarum. The quantification of CPDs was performed by ELISA with CPD monoclonal antibody. Blue: wild type; Orange: a transgenic strain expressing HsPHR2. (PDF) [file pone.0043069.s003.pdf]

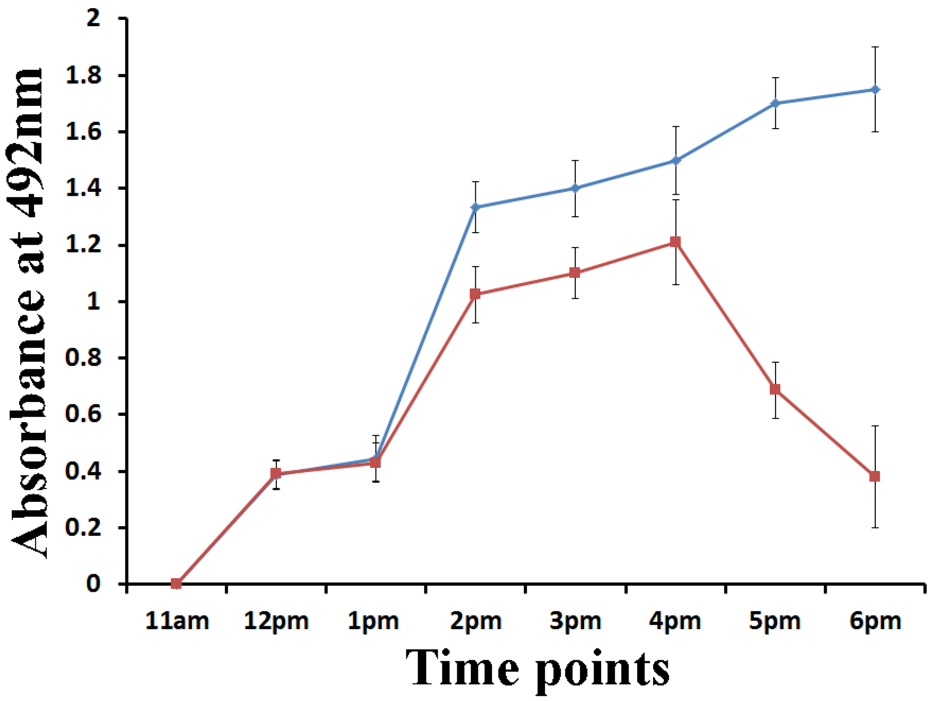


Fig. S3 The amount of CPDs in the solar-irradiated mycelium of wild type and transgenic *B. bassiana* expressing a CPD photolyase (HsPHR2) from *H. salinarum* . The quantification of CPDs was performed by ELISA with CPD monoclonal antibody. Blue: wild type; Orange: a transgenic strain expressing HsPHR2.
