# Supplementary material for: Enhanced UV Resistance and Improved Killing of Malaria Mosquitoes by Photolyase Transgenic Entomopathogenic Fungi
Source: PLoS One. 2012 Aug 17;7(8):e43069. doi: 10.1371/journal.pone.0043069 (PMC3422317; doi:10.1371/journal.pone.0043069)
Supplement: Figure S4 — Kinetics of mosquito survivorship in bioassays. Female adult mosquitoes (A. gambiae) were sprayed with B. bassiana spore suspensions (1×10^7^ spores/mL) that were irradiated by sunlight for 4 h. Red = the wild type strain; Blue = a transgenic strain expressing a CPD photolyase (HsPHR2) from H. salinarum; black = control insects that were treated with 0.05% Tween-80. (PDF) [file pone.0043069.s004.pdf]

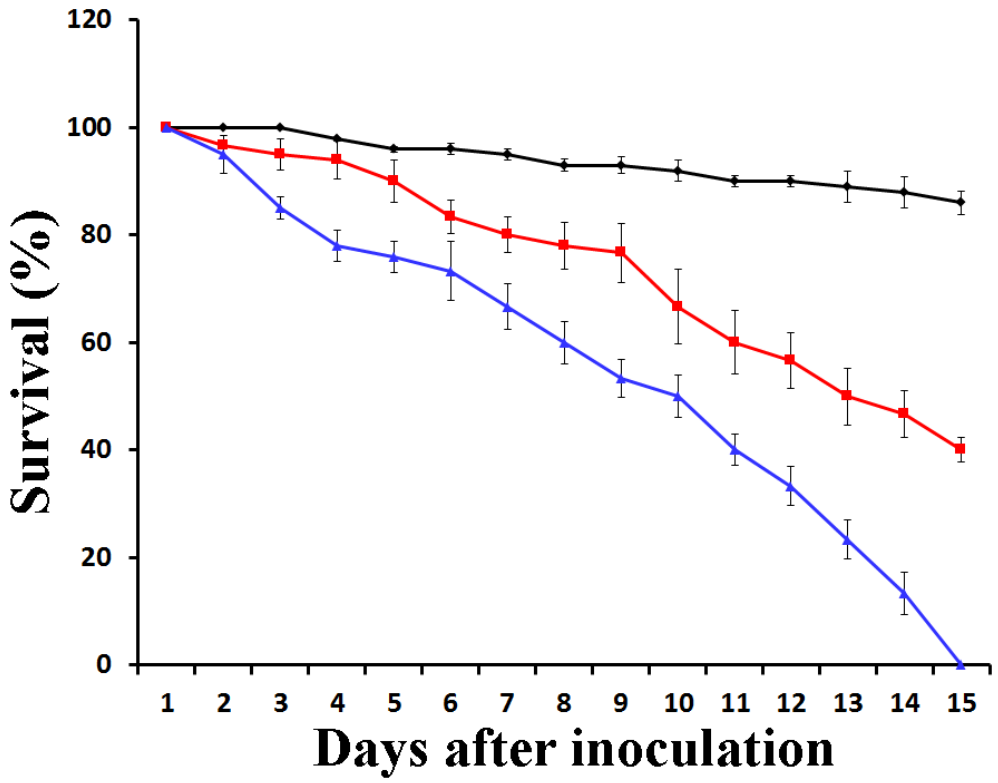

Fig. S4. Kinetics of mosquito survivorship in bioassays. Female adult mosquitoes (*A. gambiae*) were sprayed with *B. bassiana* spore suspensions (1×10^7 spores/mL) that were irradiated by sunlight for 4h. Red=the wild type strain; Blue= a transgenic strain expressing a CPD photolyase (HsPHR2) from *H. salinarum*; black= control insects that were treated with 0.05% Tween-80.
