# Supplementary material for: Enhanced UV Resistance and Improved Killing of Malaria Mosquitoes by Photolyase Transgenic Entomopathogenic Fungi
Source: PLoS One. 2012 Aug 17;7(8):e43069. doi: 10.1371/journal.pone.0043069 (PMC3422317; doi:10.1371/journal.pone.0043069)
Supplement: Table S1 — The ability to repair CPDs and (6-4)PPs of ΔMrphr1, ΔMrphr2, ΔMrphr1ΔMrphr2 and the wild type irradiated by UV followed by either photoreactivation or NER. (PDF) [file pone.0043069.s005.pdf]

Table S1 The ability to repair CPDs and (6-4)PPs of *ΔMrphr1*, *ΔMrphr2*, *ΔMrphr1ΔMrphr2* and the wild type irradiated by UV followed by either photoreactivation or NER[1]

CPDs (OD_{492nm}) [2]				(6-4)PPs (OD_{492nm}) [2]			
WT	*ΔMrphr1*	*ΔMrphr2*	*ΔMrphr1ΔMrphr2*	WT	*ΔMrphr1*	*ΔMrphr2*	*ΔMrphr1ΔMrphr2*
No photoreactivation and no NER							
2.46±0.21	2.07±0.13	2.49±0.08	2.26±0.22	2.35±0.14	2.27±0.2.4	2.47±0.16	2.33±0.14
Photoreactivation (4h-exposure to fluorescent light bulbs)							
0.985±0.11	2.07±0.23	1.10±0.21	2.36±0.13	1.56±0.18	1.61±0.12	2.38±0.07	2.35±0.15
NER (incubation in the dark)							
1.58±0.09	1.84±0.15	1.61±0.17	1.96±0.04	1.66±0.11	1.74±0.03	1.69±0.12	1.88±0.08

Note:

1. Mycelium suspended in 0.01% yeast extract was irradiated UV (8mW/cm^2) followed by either photoreactivation [4h-exposure to two fluorescent lights bulbs (15W, Sylvania F15T8/CW/SS) or NER (incubation in the dark for 6h).
2. 10ng of DNA was used for CDP quantification by ELISA, and 400ng for (6-4)PPs.
