# Supplementary material for: Enhanced UV Resistance and Improved Killing of Malaria Mosquitoes by Photolyase Transgenic Entomopathogenic Fungi
Source: PLoS One. 2012 Aug 17;7(8):e43069. doi: 10.1371/journal.pone.0043069 (PMC3422317; doi:10.1371/journal.pone.0043069)
Supplement: Table S2 — The ability to repair CPDs of wild type and transgenic M. robertsii and B. bassiana radiated by UV followed by photoreactivation. (PDF) [file pone.0043069.s006.pdf]

Table S2 The ability to repair CPDs of wild type and transgenic *M. robertsii* and *B. bassiana* radiated by UV followed by photoreactivation[1]

Strains	Time for photoreactivation (the value is the absorbance at 492nm)[2]				
	0h	1h	2h	3h	4h
WT-Mr	3.04±0.05	2.06±0.11	1.40±0.02	1.29±0.03	1.18±0.14
Mr-OMrPhr1	3.10±0.17	1.44±0.13	1.22±0.06	1.01±0.08	0.41±0.09
Mr-HsPhr1	3.09±0.11	1.05±0.07	0.71±0.09	0.37±0.11	0.17±0.07
Bb-WT	3.82±0.13	3.52±0.15	2.84±0.18	2.61±0.12	2.44±0.13
Bb-HsPhr1	3.88±0.14	3.25±0.17	2.35±0.11	2.02±0.18	1.06±0.09

Note:

1. Mycelium suspended in 0.01% yeast extract was irradiated UV (30mW/cm^2) followed by either photoreactivation [exposure to two fluorescent lights bulbs (15W, Sylvania F15T8/CW/SS)].
2. 10ng of DNA was used for CPD quantification by ELISA assay.
