# Supplementary material for: Enhanced UV Resistance and Improved Killing of Malaria Mosquitoes by Photolyase Transgenic Entomopathogenic Fungi
Source: PLoS One. 2012 Aug 17;7(8):e43069. doi: 10.1371/journal.pone.0043069 (PMC3422317; doi:10.1371/journal.pone.0043069)
Supplement: Table S3 — The sequences of primers and synthesized genes used in the study. (PDF) [file pone.0043069.s007.pdf]

Table S3. The sequences of primers and synthesized genes used in the study

| Name | DNA sequence | Note |
|---|---|---|
| DMrphr15-1 | TCTAGATCCGATAGCTTGGTGACC | Disruption of MrPhr1 |
| DMrphr15-2 | TCTAGATGCGCGGAGCATGAAATG | Disruption of MrPhr1 |
| DMrphr13-1 | TCTAGATTGGGACTTGGATCATTC | Disruption of MrPhr1 |
| DMrphr13-2 | TCTAGATGATTTCTCCGTAGGAGG | Disruption of MrPhr1 |
| DMrphr1CF1 | ATCACAAAGATTGTAGAG | Confirmation of disruption of MrPhr1 |
| DMrphr1CF2 | CTTATCAGGATCATTCTC | Confirmation of disruption of MrPhr1 |
| DMrphr25-1 | TCTAGACGACACTGGGTGGAAAGG | Disruption of MrPhr2 |
| DMrphr25-2 | TCTAGATACAACTTTTTGATTGCC | Disruption of MrPhr2 |
| DMrphr23-1 | TCTAGACCAATGTTTGACTTTGGG | Disruption of MrPhr2 |
| DMrphr23-2 | TCTAGACGTTAGACGTGCTCTATC | Disruption of MrPhr2 |
| DMrphr2CF1 | CGGAGCTGAAGAATTTGG | Confirmation of disruption of MrPhr2 |
| DMrphr2CF2 | CAATGCCAGATTAAAACC | Confirmation of disruption of MrPhr2 |
| Mrphr1ORF5 | GGATCCATGGCTCGAAAATCATC | Overexpression of MrPHr1 |
| Mrphr1RF3-his | CTCGAGCTAGTGGTGATGGTGATGATGCATGCCATTGGCG | Overexpression of MrPHr1 |
| NLS-GFP-5 | GGATCCATGGACAAGAAGCGCAAGTCCTGGGGTCAGGTCCTCCCCGAGCC<br>CAAGACCAACCTCCCCCCCCGCAAGCGCGCCAAGACCGATGGTGAGCAAG<br>GGC | Construction of nls:gfp |
| NLS-GFP-3 | GATATCTTACTTGTACAGCTCGTC | Construction of nls:gfp |
| NLS-HsPHR2 | GGATCCATGGACAAGAAGCGCAAGTCCTGGGGTCAGGTCCTCCCCGAGCCCAAGACCAACCTCCCCC<br>CCCGCAAGCGCGCCAAGACCGATGCCTGCTGCTCAGCCTCCTGGTATGCAGCTGTTCTGGCACCGCC<br>GTGATCTTCGCACCACTGACAACCGTGGTCTCGCTGCTGCTGCTCCTGGTGTCACCGCCGTTGATGGT<br>GGTCACGACCAGGGTCCTGTCGCTGCTGTTTTCTGCTTCGACGATGAGGTCCTGGCTCATGCTGCTCC<br>TCCTCGTGTTGCTTTCATGCTTGACGCTTTGGCTGCTCTCCGAGAGCGTTACCGAGATCTGGGCAGCG<br>ACCTTATTGTCCGTCACGGTGACCCTGCCGCTGTCTTGCCCGCTGTTGCCAACGACCTCGATGCCACC<br>CGCGTCGTTTGGAACCATGACTACTCTGGCTTGGCTACTGACCGAGATGCTGGTGTCCGCGATGCTCT<br>CGACGCTGCTGGTGTTGCTCACGCTCAGTTCCATGACGCCGTCCACCATCGTCCTGGCGAGATCCGAA<br>CCAACGCTGGCGACCCCTACTCTGTCTACACTTACTTCTGGCGCAAGTGGCAGGACCGTGAGAAGAA<br>CCCTCCTGCTCCTGAGCCTGAGCCTGCTGATCTGGCCGCTGACACCGCTCTTGCCGACACTAGCCCTT<br>TGCCCTCTGTTCAGGAGCTCGGCTTCGCTGAGCCTGAGGCTGCTGTCCCTGACGCTGGTACCGCTGCT<br>GCTCGATCCCTCCTGGATGCTTTCCGAGAGAGCGGCGACATTTACCGTTACGAGGATCGACGCGACT<br>ACCCTCACGAGGAGCCCACCTCTCGTTTGTCCCCTCATCTCAAGTTCGGCACCATCGGTATTCGAACT<br>GTCTACGAGGCTGCTCGAGCTGCTAAGTCCGACGCCGATACTGACGATGAGCGAGAGAACGTTGCTG<br>CTTTCATCGGTCAGCTGGCTTGGCGAGAGTTCTACGCTCAGGTCCTTTACTTCAACCAGAACGTCGTT<br>AGCGAGAACTTCAAGGCCTACGAGCACCCTATCGAGTGGCGAGACGATCCTGCTGCTCTCCAGGCTT<br>GGAAGGATGGCGAGACCGGTTACCCCATTGTTGACGCTGGTATGCGTCAGCTGCGAGCTGAGGCCTA<br>CATGCACAACCGCGTCCGTATGATCGTTGCTGCCTTCCTTACCAAGGATCTTTTGGTCGACTGGCGTG<br>CCGGTTACGACTGGTTCCGAGAGAAGCTCGCCGACCACGATACTGCTAACGATAACGGTGGTTGGCA<br>GTGGGCTGCTAGCACCGGTACTGACGCTCAGCCTTACTTCCGCGTCTTCAACCCCATGACCCAGGGCG<br>AGCGTTACGACCCTGATGCCGACTACATCACTGAGTTCGTCCCTGAGCTCCGCGATGTTCCCGCTGAC<br>GCCATTCACTCTTGGCATGAGCTCTCCCTGAGCGAGCGTCGACGACACGCTCCTGAGTACCCTGATCC<br>CATCGTCGACCATTCCCAGCGTCGAGAGGATGCTATTGCTATGTTCGAGCGAGCTCGAGGCGACGAG<br>TAAGATATC | |
